# Supplementary material for: Climate-driven invasion and incipient warnings of kelp ecosystem collapse
Source: Nat Commun. 2024 Jan 9;15:400. doi: 10.1038/s41467-023-44543-x (PMC10776680; doi:10.1038/s41467-023-44543-x)
Supplement: Supplementary file 1 — Supplementary Information [file 41467_2023_44543_MOESM1_ESM.pdf]

Supplementary Information (Supplementary Tables 1 to 5)

Climate-driven invasion and incipient warnings of kelp ecosystem collapse

Scott D. Ling, John P. Keane

Corresponding author: Scott D. Ling
Email: Scott.Ling@utas.edu.au

Supplementary Table 1. Analysis of variance table for 2-factor nested ANOVA testing the effects of “Time”, i.e., fixed effect, 2001/02 *versus* 2016/17; and “Site” (i.e., random effect, sites 1-13), plus the interactive term of “Time” by “Site”, and “Subsite” nested within “Time” by “Site” on the response of (a) *Centrostephanus rodgersii* abundance, and (b) barrens cover. Transformation required to meet the assumption of homogeneity of variances is shown in square brackets. Density and cover estimates at the site level were based on means of n=3 sub-sites, with sub-site estimates themselves generated from the mean of n=4 transects surveyed by divers. Significance codes are ‘***’ <0.001, ‘**’ <0.01, ‘*’ <0.05, ‘.’ 0.1; “% var.” indicates percentage of variation explained by each source.

a. *Centrostephanus* density [log(*Y*+0.001)]

Source	Df	Sum Sq	Mean Sq	F-test Df	F value	Pr(>F)		% var.
Time	1	73.3	73.3	1,12	14.4	0.003	***	2.0
Site	12	1651.2	137.6	12,52	11.7	<0.001	***	46.1
Time*Site	12	61.2	5.1	12,52	0.4	0.94		1.7
Subsite (Time*Site)	52	612.4	11.8	52,234	2.3	<0.001	***	17.1
Residuals	234	1180.5	5.0					33.0

b. Barrens cover [log(*Y*+0.001)]

Source	Df	Sum Sq	Mean Sq	F-test Df	F value	Pr(>F)		% var.
Time	1	381.2	381.2	1,12	21.7	<0.001	***	7.9
Site	12	1761.7	146.8	12,52	8.1	<0.001	***	36.6
Time*Site	12	210.9	17.6	12,52	1.0	0.49		4.4
Subsite (Time*Site)	52	941.6	18.1	52,234	2.3	<0.001	***	19.6
Residuals	234	1513.9	6.5					31.5

Supplementary Table 2. Summary of change in *C. rodgersii* urchin barrens and kelp bed cover (including species composition) for eastern Tasmania from 2001/02 to 2016/17. Data is planar cover from 5 by 1m quadrats as scored *in situ* by SCUBA divers (6-18 m depth) and has been averaged across all sites (1-13); site means were calculated hierarchically from the mean of 3 sub-sites per site, with sub-site means calculated from mean of 4 transects per sub-site within each time period. "Difference" is the average in 2016/17 minus the average in 2001/02; "Percent change" is the "Difference" divided by 2001/02 value multiplied by 100. Thermal mid-points of the macroalgal species distributions are shown to indicate relative thermal tolerance of species to coastal warming (after Soler et al. 2022 - *Cystophora* sp. is the mean of respective midpoints for *C. platylobium* & *C. retroflexa*). Notably all macroalgae with thermal midpoints below 16°C (denoted by asterisks) declined over the 15-year period, meanwhile macroalgae with thermal midpoints >16°C all increased in cover.

	**Mean Percentage cover (%)**				
**Taxa**	**2001/02**	**2016/17**	**Difference**	**Percent change**	**Species Thermal mid-point (°C)**
Urchin barrens	1.57	6.20	4.63	+294%	
Phyllospora comosa	29.21	32.86	3.65	+13%	17.6
Ecklonia radiata	29.31	34.13	4.82	+16%	18.4
*Lessonia corrugata**	2.70	1.60	-1.10	-41%	14.5
*Durvillea potatorum**	2.07	2.03	-0.04	-2%	15.9
Acrocarpia paniculata	1.07	1.46	0.39	+36%	17.6
Cystophora sp.	0.62	1.15	0.53	+87%	16.4
Sargassum sp.	0.55	1.28	0.73	+133%	17.2
*Macrocystis pyrifera**	0.36	0.23	-0.14	-37%	14.5
Summed algal cover	65.89	74.74			

Supplementary Table 3. Analysis of variance table for 3-factor ANOVA testing the effects of "Depth", i.e. fixed effect of depth strata (levels: 4-6 m, 6-8 m, 8-10 m, 10-12 m, 12-14 m, 14-16 m, 16-18 m), "Substratum", i.e. fixed effect (levels: Flat Rock, Large Boulders, Small Boulders) and "Time", i.e. fixed effect (levels: 2001/02, 2016/17), plus all interactive terms for the response of (a) *C. rodgersii* density, and (b) urchin barrens cover, for sites 1-9 in eastern Tasmanian where barrens were recorded during both survey periods. Data are means pooled across Sites 1-9 for each combination of substrate, depth and time, see Fig. 3 for n quadrat samples for each substrate type and depth in each time period. Transformation required to meet the assumption of homogeneity of variances is shown in square brackets. Significance codes as per previous.

a. *Centrostephanus* density [$Y^{0.25}$]

Source	Df	Sum Sq	Mean Sq	F value	Pr(>F)	% var.
Substratum	2	15.1	7.55	32.34	<0.001***	18.0
Depth	6	3.0	0.51	2.17	0.046*	3.6
Time	1	2.0	2.03	8.71	0.003**	2.4
Substratum*Depth	11	2.5	0.23	0.99	0.46	3.0
Substratum*Time	2	1.1	0.54	2.3	0.10	1.3
Depth*Time	6	1.9	0.31	1.337	0.24	2.2
Substratum*Depth*Time	10	2.0	0.2	0.87	0.56	2.4
Residuals	240	56.1	0.23			66.9

b. Barrens cover [sqrt(*Y*)]

Source	Df	Sum Sq	Mean Sq	F value	Pr(>F)	% var.
Substratum	2	83.1	41.53	13.52	<0.001***	8.1
Depth	6	42.7	7.11	2.31	0.034*	4.2
Time	1	83.5	83.47	27.17	<0.001***	8.1
Substratum*Depth	11	16.5	1.5	0.49	0.91	1.6
Substratum*Time	2	27.6	13.78	4.49	0.012*	2.7
Depth*Time	6	14.3	2.39	0.78	0.59	1.4
Substratum*Depth*Time	10	20.0	2	0.65	0.77	2.0
Residuals	240	737.3	3.07			71.9

Supplementary Table 4. Multiple regression of potential explanatory variables of (a) *Centrostephanus* urchin abundance, and (b) urchin barrens cover. Data are mean abundances and percent cover respectively as assessed by divers at the 5 m^2 quadrat scale (averaged across 5 m by 1 m quadrats assessed by buddy divers on either side of the transect line), for n= 3,200 quadrat-level estimates. "*Heliocidaris*" is the native urchin *Heliocidaris erythrogramma*, "Lobster" is the spiny lobster *Jasus edwardsii*, "Abalone" is the blacklip abalone *Haliotis rubra*, Source data are provided as a Source Data file 2. Significance codes are as per previous. Column "% model var." is the percentage of model variance explained by each factor as determined using LMG estimation. Note that only explanatory variables with significant effects were retained in the most parsimonious models for (a) and (b) based on AIC ranking following stepwise 'backward' model selection.

a. *C. rodgersii* abundance, prop. var. explained by model: 12.3%

Source	Df	Sum Sq	Mean Sq	F value	Pr(>F)	% var.	Av. Coeff.
Latitude	1	641.8	641.84	195.99	<0.001***	4.7	0.54
Time	1	64.7	64.72	19.76	<0.001***	0.6	0.02
Large boulders	1	608.7	608.66	185.85	<0.001***	5.1	0.01
Depth	1	27.4	27.41	8.37	<0.01**	0.5	0.04
Heliocidaris	1	105.7	105.68	32.27	<0.001***	1.4	0.16
Lobster	1	19.8	19.80	6.05	0.01*	0.1	-0.23
Abalone	1	0	0.05	0.01	0.90	0.0	0.01
Residuals	3,192	10453.7	3.27				

b. Barrens cover, prop. var. explained by model: 61.6%

Source	Df	Sum Sq	Mean Sq	F value	Pr(>F)	% var.	Av. Coeff.
Centrostephanus	1	384691	384691	4919.28	<0.001***	55.9	5.68
Latitude	1	1462	1462	18.70	<0.001***	2.5	3.87
Time	1	3498	3498	44.72	<0.001***	1.1	0.25
Large boulders	1	73	73	0.94	0.33	0.5	0.06
Depth	1	526	526	6.73	<0.01**	0.2	0.43
Heliocidaris	1	1036	1036	13.25	<0.001***	0.1	0.51
Lobster	1	27	27	0.34	0.56	0.1	-1.31
Abalone	1	498	498	6.37	<0.05*	0.0	-0.26
Residuals	3,191	249538	78				

Supplementary Table 5. Analysis of variance table for 2-factor nested ANOVA testing the effects of “Time”, i.e. fixed effect of 2001/02 versus 2016/17; and “Site” (sites 1-9, i.e. random effect), plus the interactive term of “Time” by “Site”, and “Subsite” nested within “Time” by “Site” on the response of percentage of reef containing continuous urchin barrens (a) and decreasingly sized incipient urchin barrens (b-d), plus the estimate of planar urchin barrens cover obtained by summing the proportions of all barrens types (e). Estimates at the site level are based on means of n=3 subsites, while estimates at the subsite level are based on the means of n=4 transects. Significance codes as per previous.

	Source	Df	Sum Sq	Mean Sq	F-test Df	F value	Pr(>F)	% var.
a. Continuous Barrens	Time	1	20.9	20.94	1,8	10.6	0.012*	0.8
(log(Y+0.001)	Site	8	1366.3	170.78	8,36	17.4	<0.001***	52.7
	Time*Site	8	15.8	1.97	8,36	0.2	0.99	0.6
	Subsite (T*S)	36	353	9.8	36,160	1.9	<0.01**	13.6
	Residuals	160	838	5.24				32.3
b. Incipient - large	Time	1	12.8	12.8	1,8	21.7	0.002**	7.9
($Y^{0.25}$)	Site	8	61.9	7.7	8,36	7.6	<0.001***	38.1
	Time*Site	8	4.7	0.6	8,36	0.6	0.79	2.9
	Subsite (T*S)	36	36.8	1.0	36,160	3.5	<0.001***	22.6
	Residuals	160	46.4	0.3				28.5
c. Incipient - medium	Time	1	19.7	19.7	1,8	19.3	0.002**	11.9
($Y^{0.25}$)	Site	8	41.7	5.2	8,36	5.2	<0.001***	25.2
	Time*Site	8	8.1	1.0	8,36	1.0	0.44	4.9
	Subsite (T*S)	36	36.0	1.0	36,160	2.7	<0.001***	21.8
	Residuals	160	59.8	0.4				36.2
d. Incipient - small	Time	1	82.4	82.4	1,8	25.4	<0.001***	49.8
($Y^{0.25}$)	Site	8	17.6	2.2	8,36	3.4	0.005**	10.7
	Time*Site	8	26.0	3.2	8,36	5.1	<0.001***	15.7
	Subsite (T*S)	36	23.1	0.6	36,160	1.2	0.21	14.0
	Residuals	160	84.4	0.5				51.1
e. Planar %	Time	1	1060.1	1060.1	1,8	32.42	<0.001***	28.7
($Y^{0.25}$)	Site	8	811.2	101.4	8,36	8.38	<0.002**	22.0
	Time*Site	8	261.6	32.7	8,36	2.70	0.02*	7.08
	Subsite (T*S)	36	434.9	12.1	36,160	1.72	0.01*	11.8
	Residuals	160	1125.1	7.0				30.5

References

1. Soler, G.A., Edgar, G.J., Barrett, N.S., Stuart-Smith, R.D., Oh, E., Cooper, A., Ridgway, K.R. and Ling, S.D., 2022. Warming signals in temperate reef communities following more than a decade of ecological stability. *Proceedings of the Royal Society B*, **289**, 20221649.
